# A Randomized Clinical Trial of the Efficacy of KID21 Point (Youmen) Acupressure on Nausea and Vomiting of Pregnancy

**DOI:** 10.5812/ircmj.2153

**Published:** 2012-11-15

**Authors:** Mojgan Naeimi Rad, Minoor Lamyian, Reza Heshmat, Mohammad Asghari Jaafarabadi, Shahla Yazdani

**Affiliations:** 1 Department of Reproductive Health and Midwifery, Medical Science Faculty, Tarbiat Modares University, Tehran, Iran; 2 International College of Acupressure Medicines, Lyon, France; 3 Departments of Statistics and Epidemiology, Faculty of Health and Nutrition, Tabriz University of Medical Sciences, Tabriz, Iran; 4 Departments of Obstetrics and Gynecology, Babol University of Medical Sciences, Babol, Iran

**Keywords:** Acupressure, KID21 Acupoint, Nausea, Pregnancy, Vomiting

## Abstract

**Background:**

Nausea and vomiting in pregnancy is a common complaint of nearly 50-80% of pregnant women. The problem begins around the 4th weeks of pregnancy and often stays up to the 12th weeks and may continue to the 16th week in a few patients.

**Objectives:**

The aim of our study is to determine the effect of acupressure (on KID21 point) on nausea and vomiting of pregnancy.

**Materials and Methods:**

This single blind clinical trial study was performed on 80 women with nausea and vomiting in the first trimester of pregnancy. Women were randomly divided to two groups; study group with the acupressure on KID21 point and the placebo group with pressure on sham acupressure for 20 minutes per day in four consecutive days. The intensity of nausea was assessed by visual Analogue scale (VAS) and vomiting frequency was evaluated by counting during these four days. Then the results compared with each other.

**Results:**

The intensity of nausea and vomiting between two groups on the fourth day was shown differences (P<0.001).

**Conclusions:**

Acupressure on KID21 point is more effective than sham acupressure in reduction of nausea and vomiting in pregnancy.

## 1. Background

Nausea and vomiting in pregnancy is a common complaint of nearly 50-80% of pregnant women (1). The problem begins around the 4th weeks of pregnancy and often stays up to the 12th weeks and may continue to the 16th week in a few patients (2). But in Laroix study showed that "Morning sickness" occurred in only 1.8% of women, whereas 80% reported nausea lasting all day. Only 50% of women were relieved by 14 weeks of pregnancy and 90% relief by 22th week pregnancy (1). Since, nausea and vomiting of pregnancy has a great impact on the quality of life of pregnant women, should not be left untreated even in normal condition (3). In recent years use of medicines was diminished and often non-medication therapies and complementary and alternative medicines (CAM) are preferred (4). A study as "the pregnant women decision to receive medication to reduce nausea and vomiting" showed 34% of pregnant women did not use the medicine (vitamin B6) and 26% used less than prescribed doses and they expressed it for the lack of trust in medication safety in pregnancy and preferring CAM (5). Out of CAM, acupressure is a kind of stimulation to the acupuncture points with finger pressure or use hand. Their experts believe that this pressure cause different effects according to different parts stimulation (6). Acupressure widely used in the treatment of nausea and vomiting in traditional medicine in China. No mechanism of acupressure in the treatment of nausea and vomiting of pregnancy was known exactly. According to possible mechanism theory, acupressure by creating pressure on specific points are activated the small myelin nerves in the muscles and pass stimulations to the higher nerve centers, including spinal cord, midbrain, hypothalamus and pituitary axis. Thus, different effects are demonstrated depending on the location of stimulation (7).

**Figure 1:**
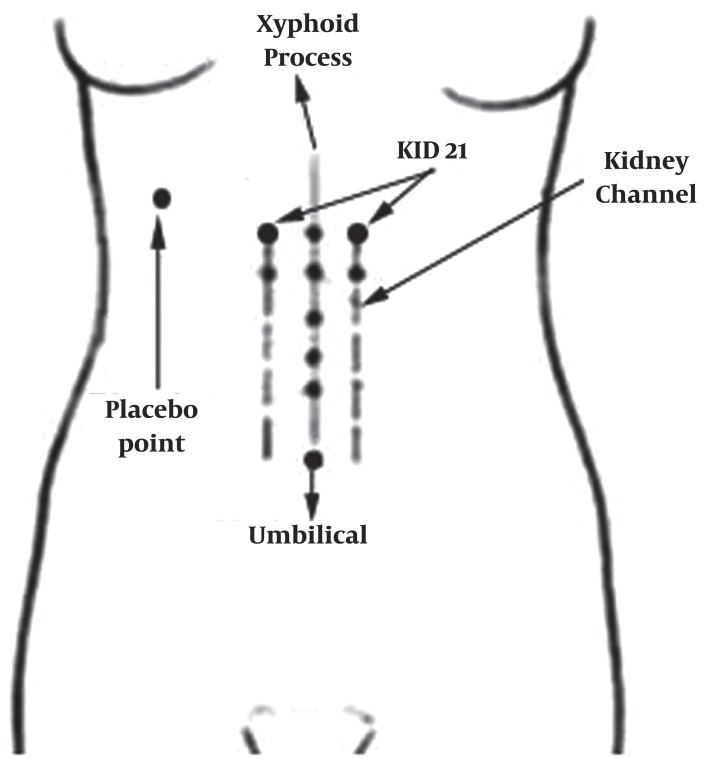
The Youmen and the Placebo acupoints

Due to the internal connection that exists between the stomach and the uterus via the Penetrating vessel any rebellious qi within the Penetrating vessel has the potential to interfere with the descending action of the stomach, creating nausea and vomiting. A practical way to harmonize the Penetrating vessel is to utilize KID21,(8) that's why we chose this point. One of the points which are applied in the treatment of nausea and vomiting of pregnancy is the KID21 point which located on the kidney channel, 6 CUN (A traditional Chinese unit of measure that is the width of a person's thumb at the knuckle) above the navel and 0.5 CUN on both sides of midline as the two symmetric points ( Figure 1 ).

Pressure on this point can cause recovering nausea and vomiting of pregnancy for who are unable to use their hands, due to dysfunction and disability such as swelling, burn, dermatitis and fracture hands. Other treatment indications of this point included: abdominal pain, chest pain, coughing blood, blood in stool, diarrhea, women heart pain, memory deficit, breast abscess, and lack of breast milk flow (9). Acupressure associates with much benefit of the physical comfort and satisfaction and also cost-effective (10). Moreover, its learning and application is so simple without negative side effects (even if partially performed). It also reduces stress in the patients (11). As one of the prenatal cares, controlling nausea and vomiting during pregnancy is often a midwife challenge, research on CAM may offer more useful ways to solve this common problem of pregnancy.

## 2. Objectives

Our study is the first research that was designed to evaluate the effect of acupressure on KID21 point alone versus sham acupressure on nausea and vomiting of pregnancy.

## 3. Materials and Methods

This clinical trial single-blind study was performed on 80 pregnant women with nausea and vomiting of pregnancy in clinic prenatalogy of Rouhani Hospital of Babol University of Medical Science from Jan to June 2011. Also, This study approved by ethical committee of Tarbiat Modares University of Tehran. We calculated the sample size based on a similar study by Ozgoli, 12 using the following formula:

n= [Z _(1-α/2)_ + Z _(1-β)_] ^2^ × {σ1^2^ + σ2^2^} / (d) ^2^ with a = 0.05 and power= 90%.We concluded that each group should consist of at least 40 pregnant women. 40 pregnant women in intervention group and 40 in placebo group who divided according to block randomization method in a block of six. Pregnant women were informed of the purpose of the study and told that they could drop out at any time, after they gave written consent. Inclusion criteria were healthy pregnant women in age 18-35 yr, singleton pregnancies, the first trimester of pregnancies, moderate to severe nausea and vomiting, normal electrolytes, lack of diseases causing nausea and vomiting such as gastrointestinal disease, normal blood pressure and lack of ketonuria, unwanted pregnancies, passive or active smokers, as well as avoidance of effective drug on nausea and vomiting .Exclusion criteria: women without tendency to go on the study or who were lose to follow-up. Then gestational age, intensity of nausea and frequency of vomiting were matched in these women.

The data gathering tool was a questionnaire. First, they also completed a questionnaire about their sociodemographic characteristics, and the intensity of nausea was assessed using the standard VAS (visual analogue scale) scale (12). This visual tool includes a 10 cm ruler with certain beginning and end area and clear range that patients indicate their health condition on it. Zero represents the best condition (lack of nausea) and number 10 represents the worst (severe nausea). This visual scale of nausea intensity record is self-report. As the nausea is felt by patient, self-report technique is appropriate for nausea measurement. Also in this study, frequency of vomiting in each day is used to evaluate the intensity of vomiting.

### 3.1. Processing 

In both groups routine tips to reduce nausea and vomiting include: increasing meal, eating smaller portions of food, giving up food before fullness, avoiding fatty and spicy foods and eat crackers or dry bread before arising of sleep, being hydrated was learned by educational pamphlets. Then, the pregnant women were reminded voluntary participation in this study and finally the informed consent were signed by them. It is noteworthy that whole of the study processes were done by the same researcher that had certificate to doing this procedure. All pregnant women have taken vitamin B6 (40 mg BD) and were asked to refer in 5:00 PM and 7:00 PM. The windows and the door were kept closed and no one was allowed to enter the room to prevent stimuli. Then they were placed in supine position. Before applying acupressure, desired points in both groups were confirmed by Acuhealth Tens pro 900 device. (Acuhealth Pty, Stepney, South Australia) The researcher gently placed her thumb on the two symmetrical KID21 points in the study group and on a false point in the placebo group and gradually increased the pressure as much as no pain existed. If the pain appeared, before continuing, we stop the pressure till the pain disappeared totally. At this time, it was used Force Gauge (Lurton Electronic Enterprise Co., Ltd, Taipei, Taiwan) to apply matched pressure and displayed pressure rate was recorded by the system. Nail color changing caused by pressure was used as pressure scale. The Pressure was done two minutes on both point KID21 which were according to clockwise for one minute and unlike it for another minute. The pressure was strong and deep, but as much as the tolerance of patients. Then the pressure was ceased and only massage of the point continued for two more minutes to stimulate meridian and it was repeated for 20 minutes as above method and performed similarly for four consecutive days. Also, in the placebo group, the pressure was similarly applied on the false point (a lack of energy point) for 20 minutes daily for four consecutive days by the researcher. The pregnant women were daily recorded changes of nausea intensity during these four days and filled VAS (Visual Analogue Scale) questionnaires. Moreover, frequency of vomiting was recorded in both groups every day to evaluate intensity of vomiting. The pregnant women were told that they could apply this acupressure protocol whenever they felt nausea and vomiting and were taught how to pressure on KID21 point.

Data was analyzed by SPSS version 16(SPSS,Chcago,IL,USA).To compare the demographic criteria of the two groups, Chi-square and T tests and to compare intensity of nausea and frequency of vomiting was used Mann-Whitney, Friedman and Sign-rank test. P<0.05 was considered significant.

## 4. Results

Out of 85 pregnant women were participated in our study, 3 patients in the intervention group and 2 patients in the placebo group did not go on study, so they were excluded. Among 80 pregnant women with nausea and vomiting of pregnancy that completed the study, no significant differences were seen according to age, gestational age, education, occupation, house owner or tenant, BMI, gravid, parity, abortion and, serum sodium and potassium levels before Pressure. There were also no significant differences in the intensity of nausea and frequency of vomiting in each group before entering the study and Pressure tone applied for acupressure was not statistically different between the two groups (Table 1).

**Table 1:** Characteristics of the 80 study participants

Criteria	Intervention group (n=40) a	Placebo group (n=40)	P value
**Age (year)**	26.03 ± 4.18	25.88 ± 5.58	0.892
**Education(year)**	11.7 ± 3.19	12.18 ± 3.03	0.498
**BMI**	24.39 ± 4.07	25.64 ± 5.14	0.234
**Life place ownership**			0.056
Yes	31(57.4%)	23(42.6%)	
NO	9(34.6%)	17(56.4%)	
**Gestational age (week)**	9.55 ± 1.81	9.45 ± 2.02	0.817
**Gravity**	2(2-1)	1(2-1)	0.075
**Parity**			0.065
Nulli	21(52.5%)	29(72.5%)	
Multi	19(47.5%)	11 (27.5%)	
**Abortion**			0.775
Yes	8(20%)	7(17.5%)	
No	32(80%)	33(82.5%)	
**Occupation**			0.446
Yes	9(22.5%)	12(30%)	
No	31(77.5%)	28(70%)	
**Sodium(mmol/l)**	138.15 ± 4.72	138.35 ± 3.62	0.782
**Potassium(mmol/l)**	4.01 ± 0.34	3.96 ± 0.31	0.530
**Nausea Intensity before pressuring**	8(10-7)	8 (9-7)	0.126
**Vomiting Intensity before pressuring**	2(4-1)	3-1))2	257.0
**Tone of pressure (mmHg)**	1040.35 ± 148.47	1073.58 ± 169.18	0.353

^a^Values are given as number (percentage) or mean ± SD or median (IQR) unless otherwise indicated

Out of 80 pregnant women with nausea and vomiting of pregnancy were 38 multigravid pregnancy that 24 patients (63.2 %) experienced nausea and vomiting in their previous pregnancy and 14 (36/ 8 %) did not that. The intensity of nausea on the fourth day had difference between intervention and placebo groups (P<0.001, U =228.5) ( Table 2 ), also, the frequency of vomiting on the fourth day between intervention and placebo groups had differences (P<0.001, U=380) ( Table 3 ). The intensity of nausea and frequency of vomiting during the acupressure days was showed difference within each groups (P<0.001) and the intensity of nausea and frequency of vomiting between before pressure and the fourth day was also showed difference in the two groups (P < 0.001). No side effect of this intervention was found in our study.

**Table 2:** Comparison of intensity of nausea from the first day to the fourth day of acupressure in two groups under study

Nausea intensity after pressuring	Intervention group (n = 40)^a^	Placebo group (n = 40)	P Value
**1st day**	7(8-6)	7(8-6)	0.473
**2stday**	6(7.75-4)	7(8-6)	0.012
**3st day**	5(5-3)	7(8-5)	< 0.001
**4st day**	4(5-2)	7(8-5)	< 0.001

^a^Values are given median (IQR). By the mann-whitney U test

**Table 3:** Comparison of intensity of vomiting from the first day to the fourth day of acupressure in two groups under study

Vomiting intensity after pressuring	Intervention (n = 40)^a^	Placebo (n = 40)	P Value
**1st day**	1 (2-0)	1 (2-1)	0.012
**2st day**	0 (1-0)	1 (2-0.25)	0.003
**3st day**	0 (1-0)	1 (2-0)	0.001
**4st day**	0 (0.75-0)	1 (2-0)	< 0.001

^a^Values are given median (IQR).By the mann-whitney U test

## 5. Discussion

This study was shown pressure on KID21 point in intervention group is more effective than placebo (sham acupressure) group in treatment of nausea and vomiting during the first trimester of pregnancy. Many studies have emphasized effectiveness and safety of acupressure in treatment of nausea and vomiting during pregnancy (13). We Searched internet and magazines but we found no article published about pressure effect of KID21 point alone in the treatment of nausea and vomiting of pregnancy. Although, Smith in Australia has used acupuncture KID21 point along with other effective points on nausea and vomiting of pregnancy and concluded acupuncture at these points improved in five quality of health versus the point P6 alone, sham acupressure and, non-treatment group. However, the nausea had been reduced, but no differences in vomiting were found. As Smith suggested frequency of the treatment may show more beneficial effects (14). In spite of using acupuncture in smith study, KID21 point has also been used like our research, however they used this point associated with other points, so making decisions on therapeutic effect of acupressure KID21 point should be done with caution. We chose to use a single point to avoid confusing our pregnant women who were to self- treat. Also Can Gurkan concluded wristband with pressure at the P6 point can have therapeutic and also psychological effects (13). This study confirm our results. The placebo effect was controlled in their study similarity of our study. But in their study, the P6 point has been used in the treatment of nausea and vomiting of pregnancy.

In another study was done by Jamigorn called "acupressure and vitamin B6 in the treatment of nausea and vomiting of pregnancy", showed that the acupressure on the P6 point in the treatment of mild to moderate nausea and vomiting of pregnancy had no advantage compared to vitamin B6 (15). But in their study, the KID21 point has not been used in the treatment of nausea and vomiting of pregnancy and has been used wristband instead of finger pressure.

In the study which Northeim and the colleagues was conducted was shown that pressure with the use of wristband on the P6 relates to wristband without pressure reduce symptoms of morning sickness. So, it can use before pharmaceutical treatment to reduce symptoms of nausea and vomiting during pregnancy (16). This study confirm our results that KID21 effect on nausea and vomiting of pregnancy. In Song study, to evaluate the effect of pressure on the P6 on ketonuria levels induced of nausea and vomiting of pregnancy concluded the pressure on P6 reduces symptoms in women with severe nausea and vomiting of pregnancy (17). In this study pregnant women had hyperemesis but they used the finger pressure similarity of our study. And also, Azgoli reported wristband without pressure is effective to decline symptoms not so much as wristband with pressure (18). That observes the placebo effects similarity of our study and confirm our results. In another study was done by Puangsricharern and Mahasukhon called" Effectiveness of Auricular Acupressure in the Treatment of Nausea and Vomiting in Early Pregnancy" Each patient in the treatment group received magnet pellets, placed at both auricles. They were taught to start acupressure from the third to the sixth day and were compared to the control group. They reported that Auricular acupressure therapy in treatment of nausea and vomiting in early pregnancy may not relieve nausea and vomiting in early pregnancy (19). This study did not confirm our study; however, the KID21 point has not been used in the treatment of nausea and vomiting of pregnancy and the placebo effect was not controlled in their study similarity of our study. Our result indicates applying acupressure on KID21 point reduce nausea and vomiting in pregnant women versus sham acupressure. We observed a significant lessening of nausea and vomiting in the placebo group, but it had not clinical importance. However, it needs further studies which exclusively emphasizing on KID21 point and compare it with the other relieving pressure points of traditional Chinese medicine or other CAM in reducing nausea and vomiting during all months of pregnancy. Maybe during these researches, a point to be found that works more effectively than others.
